# Randomized, double-blinded, controlled clinical trial of the effect of captopril, telmisartan and their combination on systemic inflammation of patients on hemodialysis

**DOI:** 10.1038/s41598-022-22656-5

**Published:** 2022-10-20

**Authors:** Susan M. Ordaz-Medina, Alfonso M. Cueto-Manzano, Juana González-Plascencia, José L. Montañez-Fernández, Elias J. Ordaz-Medina, Fabiola Martín-del-Campo, Alfonso M. Cueto-Ramírez, Petra Martínez-Martínez, Laura Cortés-Sanabria, Enrique Rojas-Campos, Benjamín Trujillo-Hernández

**Affiliations:** 1 Unidad de Investigación Médica en Enfermedades Renales, Hospital de Especialidades, Centro Médico Nacional de Occidente, Instituto Mexicano del Seguro Social, Belisario Domínguez No. 1000, Col. Independencia, CP 44320 Guadalajara, Jalisco Mexico; 2 Departamento de Nefrología, Hospital General Regional No. 110, IMSS, Guadalajara, Jalisco Mexico; 3 Facultad de Medicina, Universidad de Colima, Colima, Colima Mexico

**Keywords:** Nephrology, Renal replacement therapy, Haemodialysis

## Abstract

To evaluate individual and combined effect of captopril and telmisartan on systemic inflammation markers of hemodialysis (HD) patients. Randomized, double-blinded, controlled clinical trial. Patients on HD at least 2 months, with arteriovenous fistula, were randomly allocated to groups: (1) captopril/placebo (N 13); (2) telmisartan/placebo (N 13); (3) captopril + telmisartan (N 12); or (4) placebo/placebo (N 12). During 3 months, patients received oral drugs as follows: captopril 50 mg/day, telmisartan 80 mg/day or placebo. Patients excluded if they had conditions or were on drugs potentially influencing on inflammation. Clinical and biochemical evaluations were performed monthly. Serum tumor necrosis factor alpha (TNFα), interleukin 6 (IL-6), and C-reactive protein (CRP) were measured at 0, 1 and 3 months. Baseline, demographic, clinical and biochemical variables were comparable between groups. Baseline versus final inflammatory markers were: *captopril/placebo* TNFα, 2.47 (0.1–4.5) versus 1.73 (0.3–3.8) pg/ml; IL-6, 17.03 (7.2–23) versus 7.90 (0.7–19) pg/ml; CRP, 4.21 (1.6–18) versus 5.9 (3.0–28) mg/l; *telmisartan/placebo* TNFα, 3.03 (2.3–4.6) versus 1.70 (1.2–2.0) pg/ml; IL-6, 14.10 (5.5–23) versus 9.85 (6.2–13) pg/ml; CRP, 5.74 (2.1–13) versus 10.60 (1.5–27) mg/l; *captopril* + *telmisartan* TNFα, 1.43 (0.7–5.4) versus 0.40 (0.1–2.1) pg/ml; IL-6, 10.05 (4.9–23) versus 4.00 (0.7–7.7) pg/ml (*p* < 0.05); CRP, 3.26 (0.7–12) versus 2.83 (0.6–6.5) mg/l; *placebo/placebo* TNFα, 3.13 (1.6–5.6) versus 1.64 (1.6–2.3) pg/ml; IL-6, 8.12 (5.4–16) versus 7.60 (2.4–15) pg/ml; CRP, 5.23 (1.9–16) versus 3.13 (1.5–18) mg/l. Monotherapy with captopril or telmisartan display a trend, but their combined treatment significantly decreased serum levels of IL-6. No remarkable changes on TNFα and CRP were observed.

## Introduction

Globally, the prevalence of chronic kidney disease has been reported about 9%, and in this kind of patients, risk factors such as diabetes and cardiovascular disease contributed to more than half the deaths in 2017^1^.

On the other hand, inflammation is highly prevalent in patients with end-stage kidney disease (ESKD) on dialysis, and has been associated with multiple factors such as malnutrition, overhydration, bioincompatibility of hemodialysis (HD) membranes and dialysate, uremia, dialysis vintage, dialysis dose, and vascular access, among others^2–7^. Moreover, a strong association has been shown between inflammation and atherosclerosis^8^; thus, inflammation has been implicated in the higher cardiovascular mortality of this kind of patients^1,2^.

On the other hand, angiotensin II increases vascular inflammation, induces endothelial dysfunction, and increases atherosclerosis^9^, and has been shown to increase the production of interleukin 6 (IL-6) and tumor necrosis factor alpha (TNFα) in glomeruli, tubules and vessels in rat kidney^10^. In turn, angiotensin converting enzyme inhibitors (ACEIs) reduced TNFα, nuclear factor kappa B (NF-κB) and IL-6, in in vitro and in vivo studies^11–13^; angiotensin receptor blockers (ARBs) also suppressed chemokines and cytokines in rats^14,15^. In humans, ACEIs decreased serum TNFα and C-reactive protein (CRP) levels in patients with impaired cardiac function^16^ or coronary artery disease^17^. In an open crossover study with type 2 diabetes mellitus without kidney disease, ramipril, telmisartan, and particularly their combination, significantly decreased CRP compared to baseline^18^.

In patients with kidney disease, both ACEIs and ARBs have been extensively studied as antihypertensive and nephroprotective drugs; however, they have been scarcely studied as anti-inflammatory drugs. Our group has previously shown in a double-blinded, controlled clinical trial in patients on HD^19^, that enalapril did not significantly decrease serum levels of TNFα, IL-6 and CRP compared to placebo. Notwithstanding, current data are inconclusive and limited in this regard, particularly considering whether the chronic use of ACEIs, ARBs, or their combination, have a role in reducing inflammation in dialysis patients.

Therefore, the present study was aimed to evaluate the individual and combined effect of captopril and telmisartan on systemic inflammation markers of patients on HD.

## Methods

The present is a double-blinded, controlled and randomized clinical trial performed in patients from the HD Unit of the Hospital General Regional No. 110, Instituto Mexicano del Seguro Social (IMSS). Fifty patients on HD at least 2 months, with arteriovenous fistula as vascular access, were included.

We excluded patients with ESKD of inflammatory cause, infectious disease 2 months before the study or who were on treatment with antibiotics, pregnancy, cancer, liver disease, hypotension, AIDS, failed kidney graft, known hypersensitivity to captopril or telmisartan, and treatment with statins, immunosuppressive drugs, steroids, pentoxifylline, non-steroidal anti-inflammatory drugs, ACEIs or ARBs within four months previous to the study.

Once patients met selection criteria and granted their written informed consent, they were randomly assigned (by computer-generated randomization list) to one of the following groups: Captopril + Placebo, Telmisartan + Placebo, Captopril + Telmisartan, and Placebo + Placebo. During a period of three months, patients received the following oral drugs: captopril 50 mg/day, telmisartan 80 mg/day, or placebo (identical starch tablets).

All patients had HD with the same characteristics: 3 sessions per week, with a single-use dialyzer membrane of cellulose triacetate (Nipro Corporation, Osaka, Japan) and dialysate fluid (calcium 2.5 mEq/L, bicarbonate 35 mEq/L, and potassium 2 mEq/L). The ultrafiltered dialysate was monthly checked by triplicate for at least 3 days, using agar plates at 37 °C, and results were reported 0.1 CFU/mL throughout the study.

Patients received the assigned treatment and had monthly clinical evaluations, for a 3-month period. At the 0, 1 and 3-month visits, a mid-week sample of blood was withdrawn at the beginning of HD session for the following measurements: glucose, urea, creatinine, albumin, electrolytes, lipids, complete blood count and inflammation markers (TNFα, IL-6 and CRP).

TNFα and IL-6 were evaluated with human high sensitivity ELISA Kits, (*Invitrogen,* USA) and CRP by nephelometry with high sensitivity kits (*Dade Behring,* Germany) using a Nephelometry Analyzer II (*Dade Behring*, Germany). In the case of any infectious event developed during the study, blood samples and evaluations were postponed until 3 weeks after complete resolution.

Dialysis dose was monitoring by equilibrated Kt/Vurea^20^ at months 0 and 3. All laboratory determinations, including those of inflammation markers, were done by the same personnel of the Laboratorio Central, Hospital de Especialidades. All participants in the study (patients, personnel and investigators, including persons responsible for data management and statistics), were blinded to the treatment assignment. We opened the code only after data collection of all recruited patients were completed. Treating nephrologists were free to add or modify the dose of antihypertensive drugs different from ACEIs and ARBs, if required.

For evaluation of treatment compliance, the tablets left in the container were counted at the end of each monthly visit. The study ended when the last recruited patient finished the follow-up period.

The protocol adhered to the Declaration of Helsinki and was approved by the Local Ethics and Research Committee (Comité Local de Ética e Investigación en Salud, Hospital de Especialidades, CMNO, No. R-2009-1301-86). It was also registered in the US National Institutes of Health ICMJE Clinical Trials Registration (NCT01271478, date 06/01/2011).

### Statistical analysis

To calculate a priori the sample size of our study, we considered as significant a reduction ≥ 25% on CRP found in previous studies in our setting using other drugs (pentoxifylline^21^ and pravastatin^22^); we also took in account the in vitro effect of captopril on TNFα found in other study^11^. With the previous data, and using a formula to test proportion differences in clinical trials^23^ (with a confidence level 80%, alpha 0.05, and considering 20% of possible losses to follow-up), sample size was finally calculated as 12 subjects per group. Results are shown as mean ± SD or median (percentiles 25–75%) for dimensional variables, and as number or percentages in nominal variables, as appropriate. Data distribution was tested by the Kolmogorov–Smirnov test. Due to the sample size and heteroscedastic variance of our results, inter-group comparisons were analyzed by Kruskal Wallis (in the case of dimensional variables), χ^2^ or Fisher’s exact tests (in the case of nominal variables), as appropriate. Repeated-measurements on ranks ANOVA (in case of three repeated measures) or Wilcoxon tests (in case of two repeated measures) were employed for intra-group comparisons of dimensional variables. A one tailed *p* < 0.05 was accepted as significant, but the exact value is preferentially shown.

### Statement of ethics

This protocol was conducted ethically in accordance with the World Medical Association Declaration of Helsinki and was approved by the Local Ethics and Research Committe (Comité Local de Ética e Investigación en Salud, Hospital de Especialidades, CMNO, IMSS, No. R-2008/1301/44). Patients were included in the study after they granted informed consent.

## Results

**Figure 1:**
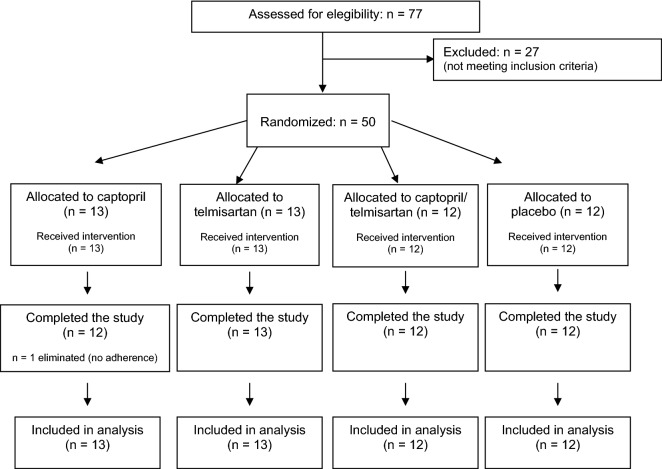
Flowchart of the clinical trial.

Fifty patients were included: 13 in the group on Captopril + Placebo, 13 in group on Telmisartan + Placebo, 12 in group on Captopril + Telmisartan, and 12 in group on Placebo + Placebo. One patient of group on Captopril + Placebo was eliminated because a clear non-adherence detected since the first month of follow-up; his results were similar to the other patients and were not eliminated from analysis. A flowchart summarizing recruitment and follow-up of patients is shown in Fig. 1.

There were no statistically significant differences between groups regarding demographic variables at baseline (Table 1). Adherence to treatment was not different between groups: group 1, 93%; group 2, 96%; group 3, 97%; and group 4, 96%.

**Table 1:** Comparison of baseline demographic characteristics between groups.

	Captopril	Telmisartan	Captopril + Telmisartan	Placebo	*p*
**Number of patients**	13	13	12	12	
**Age (years)**	56.1 ± 17.7	48.0 ± 11.7	47.0 ± 15.7	47.8 ± 19.2	0.31
**Sex, N (%)**					
Women/men	3 (23)/10 (77)	4 (31)/9 (69)	5 (42)/7 (58)	2 (17)/10 (83)	0.56
**Cause of ESKD, N (%)**					
Unknown	4 (31)	5 (38)	6 (51)	5 (41)	
Type 2 diabetes	4 (31)	5 (38)	4 (33)	3 (25)	
Polycystic disease	3 (22)	1 (8)	1 (8)	2 (17)	0.78
Obstructive	1 (8)	0 (0)	0 (0)	2 (17)	
Hypertension	1 (8)	2 (16)	1 (8)	0 (0)	
**HD vintage (months)**	60 (12–144)	55 (12–80)	69 (36–105)	48 (17–96)	0.28

*ESKD* end-stage kidney disease, *HD* hemodialysis.

In general, intermediate evaluations of clinical and biochemical variables were concordant with final results (3 months); therefore, to simplify data presentation, only baseline and final results are shown.

Table 2 shows comparisons of clinical and dialysis-related variables between groups at the beginning and the end of the study. Mean systolic blood pressure, although tended to decrease in groups 2 and 3, it was above ideal values in all groups throughout the study, whereas mean diastolic blood pressure seemed to be better controlled and remained roughly the same throughout the study in all groups. No other difference in these variables was observed between groups. All patients received 3 HD sessions per week, 3 h per session, and equilibrated Kt/Vurea was within recommended parameters.

**Table 2:** Comparison of clinical and dialysis-related variables between groups at the beginning and the end of the study.

	Captopril		Telmisartan		Captopril + Telmisartan		Placebo	
	Baseline	Final	Baseline	Final	Baseline	Final	Baseline	Final
**Body mass index (Kg/m**^**2**^**)**	24.1 ± 3.6	24.0 ± 3.5	24.5 ± 5.3	24.4 ± 5.3	24.3 ± 2.9	24.2 ± 2.9	23.1 ± 2.4	23.0 ± 2.3
**Systolic blood pressure (mmHg)**	154 ± 13	157 ± 33	156 ± 27	151 ± 21	167 ± 19	161 ± 20	152 ± 24	159 ± 31
**Diastolic blood pressure (mmHg)**	75 ± 12	75 ± 17	78 ± 13	80 ± 9	85 ± 14	81 ± 9	77 ± 9	82 ± 16
**Equilibrated Kt/V**_**urea**_** (per session)**	1.1 ± 0.2	1.1 ± 0.3	1.0 ± 0.1	1.3 ± 0.8	1.1 ± 0.2	1.0 ± 0.1	1.1 ± 0.2	1.2 ± 0.3
**Ultrafiltration (L)**	2.0 ± 0.9	2.1 ± 1.0	1.9 ± 1.2	2.0 ± 1.1	2.2 ± 0.7	2.2 ± 0.8	2.1 ± 0.7	2.1 ± 0.8
**Duration of session (h)**	3.0 ± 0	3.0 ± 0	3.0 ± 0	3.0 ± 0	3.0 ± 0	3.0 ± 0	3.0 ± 0	3.0 ± 0
**Post-dialysis weight (kg)**	65 ± 7	65 ± 8	69 ± 16	68 ± 15	64 ± 11	64 ± 11	62 ± 9	62 ± 9

Results of biochemical variables are shown in Table 3. In general, all these variables were within usual values for this kind of patients and were not different between groups. Hemoglobin tended to be initially lower in patients with captopril, but this did not reach statistical significance.

**Table 3:** Comparison of biochemical variables between groups at the beginning and the end of the study.

	Captopril		Telmisartan		Captopril + Telmisartan		Placebo	
	Baseline	Final	Baseline	Final	Baseline	Final	Baseline	Final
**Hemoglobin (g/dl)**	9.8 ± 1.8	10.4 ± 1.5	10.7 ± 1.4	11.0 ± 1.6	11.0 ± 2.2	10.8 ± 1.2	10.8 ± 1.6	11.2 ± 1.8
**Glucose (mg/dl)**	87 ± 14	85 ± 7.2	91 ± 20	98 ± 45	91 ± 19	87 ± 17	94 ± 29	103 ± 31
**Cholesterol (mg/dl)**	148 ± 41	150 ± 40	139 ± 26	144 ± 28	167 ± 38	156 ± 40	148 ± 31	146 ± 36
**Creatinine (mg/dl)**	9.5 ± 2.4	9.4 ± 2.2	10.6 ± 2.9	10.0 ± 2.3	10.4 ± 2.0	9.6 ± 2.3	10.2 ± 2.4	10.0 ± 2.3
**Potassium (mEq/l)**	4.9 ± 0.7	5.1 ± 0.8	5.2 ± 0.7	5.1 ± 0.6	5.3 ± 0.6	5.6 ± 0.9	5.4 ± 0.9	5.1 ± 0.6

### Results of inflammation markers

Results of inflammation markers are shown in Table 4. TNFα was significantly higher at baseline in patients with telmisartan compared to those with captopril or the combined treatment; it tended to decrease in all groups until the end of follow-up, but it reached statistical significance only at the 1-month evaluation in patients of telmisartan and placebo groups. IL-6, on the other hand, progressively decreased in all groups (except the placebo group) but this was statistically significant only in the group with combined drug treatment. Compared to baseline, CRP tended to increase at final evaluation in patients of telmisartan and captopril groups, whereas it remained roughly the same in those with combined drugs; in intergroup comparison, final CRP values were significantly lower in patients under treatment with both drugs than in patients on individual drugs.

**Table 4:** Comparison of inflammation markers between groups at baseline, month 1 and month 3.

	Captopril			Telmisartan			Captopril + Telmisartan			Placebo		
	Baseline	Month 1	Month 3	Baseline	Month 1	Month 3	Baseline	Month 1	Month 3	Baseline	Month 1	Month 3
**TNFα (pg/mL)**	2.47 ^†^ (0.1–4.5)	1.77 (1.4–2.6)	1.73 (0.3–3.8)	3.33 (2.3–4.6)	1.90 * (1.3–6.1)	1.70 (1.2–2.0)	1.43 ^†^ (0.7–5.4)	1.49 (0.4–2.3)	0.40 (0.1–2.1)	3.13 (1.6–5.6)	1.02 * (0.7–1.6)	1.64 (1.6–2.3)
**IL-6** **(pg/mL)**	17.03 (7.2–23.0)	7.90 (3.7–23.0)	7.90 (0.7–19.2)	14.10 (5.5–22.8)	13.60 (8.5–19.0)	9.85 (6.2–12.6)	10.05 (4.9–23.0)	5.05 (0.6–12.2)	4.00 * (0.7–7.7)	8.12 (5.4–16.2)	10.03 (4.9–20.9)	7.60 (2.4–15.0)
**CRP** **(mg/L)**	4.21 (1.6–18.0)	3.65 (2.7–17.2)	5.37 ^£^ (2.6–18.2)	5.74 (2.1–13.2)	10.10 (5.5–14.2)	10.60 ^£^ (1.5–27.3)	3.26 (0.7–11.9)	4.31 (0.3–10.1)	2.83 (0.6–6.5)	5.23 (1.9–15.8)	4.13 (1.5–8.0)	3.13 (1.5–18.5)

^†^*p* < 0.05 versus Telmisartan in the same evaluation; *p < 0.05 versus baseline evaluation in the same group; ^£^*p* < 0.05 versus Captopril + Telmisartan in the same evaluation.

No serious adverse event was reported in any of the groups throughout the study.

## Discussion

To the best of our knowledge, this is the first randomized double-blinded, controlled clinical trial comparing the effect of the combination of captopril + telmisartan *versus* their individual treatment and placebo on systemic inflammation of HD patients. Results showed that both drugs used in combination, decreased serum levels of IL-6 more importantly than when they were used isolatedly or *versus* placebo, but no remarkable changes on TNFα or CRP were observed.

Inflammation in patients on dialysis is multifactorial and could be associated to the kidney failure per se or to the ESKD treatment modality^6,24^. In the present study, most of the patients included in all groups had an unknown cause of ESKD, which is a common finding in our setting^21,22^; however, none of them had a known inflammatory etiology. Moreover, variables related to the HD procedure that could potentially affect inflammation results^3–7,24^ were controlled, as all patients were treated with the same dialysis solution, vascular access and length of HD session. Although to a significantly lesser extent, compared with bioincompatible membranes, cellulose triacetate membranes may still activate acute-phase response and cause activation of cytokines^24^. In our study, patients in all groups were treated with the same kind of hemodialyzers (cellulose triacetate) and received dialysis dose within recommended levels. Overhydration, which has been related to inflammation^3^, was also controlled as patients in all groups had similar ultrafiltration rates, and post-dialysis weight was not different throughout the study. In addition, several comorbid conditions (arterial hypertension, obesity, and diabetes mellitus), implicated in the inflammation origin^25,26^, were not significantly different between groups. It has also been suggested that genetic factors could affect serum concentrations of IL-6^27^, and thus could have influenced on our results; however, randomization may have helped to balance this variable between groups.

In a randomized, open-label, crossover trial, 37 patients with type 2 diabetes without kidney or coronary disease, received ramipril (2.5 mg/day), telmisartan (40 mg/day) or their combination during three months; a significant decrease in CRP levels were observed in all intra- but no inter-groups comparisons^18^. In patients with ESKD on HD, the natural agent curcumin significantly decreased TNFα, IL-6 and CRP but these findings were not different compared to placebo^28^. Several years ago, in a small randomized, double-blinded, controlled clinical trial in HD, we demonstrated that enalapril did not significantly decrease serum levels of TNFα, IL-6 and CRP compared to placebo^19^. Afterwards, in other small randomized, double-blind, placebo-controlled crossover study, Gamboa JL et al.showed that with a short-time treatment (7 days), valsartan and ramipril individually lowered IL-6 levels in a blood sample drawn during the HD session^29^, but ramipril also increased IL-1β and decreased IL-10 concentrations. More recently, in other randomized clinical trial, irbersartan did not modify serum concentrations of CRP, IL-1 β, IL-6, IL-8, IL-18, and transforming growth factor-β during a 12-month study period, compared to placebo^30^. In our study, with 3-month treatment period and with measurements performed at initiation of HD sessions, captopril and telmisartan decreased IL-6 but only their combined treatment reached statistical significance. In the case of TNFα, the significant decrease in the groups with telmisartan and placebo were more probably due to the higher baseline values and a possible statistical effect of regression to the mean^31^, whereas the lower CRP levels at final evaluation in the combined drug treatment were observed in relation to a trend to increase in captopril and telmisartan groups rather than to an intragroup decrease in the combined drugs group. It has been suggested that ACEIs and ARBs may have differential effects on inflammatory response in relation to their effects on bradykinin metabolism, which in turn increases inflammation^32^. ACEIs enhance bradykinin effects by decreasing its breakdown, whereas ARBs do not. Previous studies have shown that both ACEIs and ARBs may decrease some inflammation markers, including IL-6 and CRP^18,19,29^, but this issue remains controversial^30^; moreover, there was no information whether these drugs may have an additive or synergistic effect in HD patients. In the present study, we did not find any differential effect of these drugs on inflammation markers; however, a potentiation of the decrease in IL-6 was observed with the combined use of captopril + telmisartan.

Dual inhibition of the renin–angiotensin–aldosterone system, although superior to monotherapy for blood pressure control and urine protein reduction, has been associated with increased rates of adverse events in patients with kidney disease^33^. However, very limited data exist in HD patients. In our study, no significant side effects (including hyperkalemia) were observed in all the groups.

Limitations. The presence of hidden infections (*Chlamydia pneumoniae*, *Helicobacter pylorii*, chronic periodontal disease) as cause of inflammation^34^ may be regarded as a possible limitation of this study. Although they were not investigated with a more in-depth laboratory evaluation, we clinically discarded them with meticulous physical examination and clinical chart revision; randomization could have also helped to solve this problem. We did not investigate the possible anti-inflammatory effect of certain foods, which could also be seen as a limitation, but again, randomization could have partially helped to better distribute this variable between the groups. Additionally, sample size could be considered small to find differences between groups; however, it was calculated a priori (80% confidence level, alpha 0.05) to find at least 25% reduction in serum concentrations of inflammatory markers. Finally, follow-up may seem to be short; however, according to the pharmacology of the employed drugs and results from previous studies, 3 months seem to be enough time to observe an effect on inflammation markers as those used in this study. Further studies with larger numbers of patients and cardiovascular endpoints will help to establish the clinical value of our results.

## Conclusions

In conclusion, monotherapy with captopril or telmisartan display a trend, but the combined treatment significantly decreased serum levels of IL-6. No remarkable changes on TNFα and CRP were observed.

## Data Availability

The data that support the findings of this study are not publicly available due to their containing information that could compromise the privacy of research participants but are available from the corresponding author [AMCM] upon reasonable request.
